# General decline in the diversity of the airborne microbiota under future climatic scenarios

**DOI:** 10.1038/s41598-021-99223-x

**Published:** 2021-10-12

**Authors:** Vicente J. Ontiveros, Joan Cáliz, Xavier Triadó-Margarit, David Alonso, Emilio O. Casamayor

**Affiliations:** 1 Theoretical and Computational Ecology, Center for Advanced Studies of Blanes (CEAB-CSIC), Spanish Council for Scientific Research, Accés Cala St. Francesc 14, 17300 Blanes, Spain; 2 Integrative Freshwater Ecology Group, Centre for Advanced Studies of Blanes (CEAB-CSIC), Spanish Council for Scientific Research, Accés Cala St. Francesc 14, 17300 Blanes, Spain

**Keywords:** Computational models, Biodiversity, Biogeography, Community ecology, Ecological modelling, Microbial ecology, Air microbiology, Microbial ecology, Climate-change ecology

## Abstract

Microorganisms attached to aerosols can travel intercontinental distances, survive, and further colonize remote environments. Airborne microbes are influenced by environmental and climatic patterns that are predicted to change in the near future, with unknown consequences. We developed a new predictive method that dynamically addressed the temporal evolution of biodiversity in response to environmental covariates, linked to future climatic scenarios of the IPCC (AR5). We fitted these models against a 7-year monitoring of airborne microbes, collected in wet depositions. We found that Bacteria were more influenced by climatic variables than by aerosols sources, while the opposite was detected for Eukarya. Also, model simulations showed a general decline in bacterial richness, idiosyncratic responses of Eukarya, and changes in seasonality, with higher intensity within the worst-case climatic scenario (RCP 8.5). Additionally, the model predicted lower richness for airborne potential eukaryotic (fungi) pathogens of plants and humans. Our work pioneers on the potential effects of environmental variability on the airborne microbiome under the uncertain context of climate change.

## Introduction

Changes in species geographic distributions can have strong ecological and socioeconomic consequences^1^. In the case of microorganisms, air mass circulation disperses vast amounts of individuals and interconnects remote environments. Airborne microorganisms can travel between continents^2^, survive and settle on remote environments^3^, which creates biogeographic patterns^4^. The circulation of atmospheric microorganisms exacerbates global health concerns and ecological processes such as widespread dispersal of both pathogens^5^ and antibiotic resistances^6^, cloud formation and precipitation^2^, and colonization of pristine environments^3^, respectively. Airborne microorganisms also play a role in the formation of the phyllosphere, which is one of the vastest habitats on the Earth’s surface^7^ involved in nutrient cycling^8^. Yet the study of the airborne microbiome and the factors that determine its diversity and dynamics has major knowledge gaps still^9^.

Throughout Earth's history, microbial communities have changed the climate, and climate has shaped microbial communities^10^. Microorganisms can modify ecosystem processes or biogeochemistry on a global scale, and we start to uncover their role and potential involvement in changing the climate^11^. However, the effects of climate change on microbial communities (i.e., diversity, dynamics, or distribution) are rarely addressed^12^. In the case of fungal aerobiota, its composition is likely influenced by dispersal ability, rather than season or climate^13^. Indeed, the origin of air masses from marine, terrestrial, or anthropogenic-impacted environments, mainly shapes the atmospheric air microbiome^14^. However, recent studies have shown that meteorological factors and seasonality influence the composition of airborne bacterial communities^14–16^. This evidence suggests that climatic conditions may act as an environmental filter for the aeroplankton, selecting a subset of species from the regional pool, and raises the question of the relative importance of the different factors affecting both bacterial and eukaryal aeroplankton.

In the present study, we reanalyzed a long term ecological research (LTER) study that fortnightly collected airborne microbiome samples in wet deposition (both rain and snow) on a remote high-altitude mountain in the Central Pyrenees (LTER site Aigüestortes, NE Spain) over 7 years^14^. First, we examined the effects of climatic variables and aerosols’ origin (traced back from the chemical composition of atmospheric depositions) on the diversity of atmospheric microbial communities. Then, we simulated potential future responses for the airborne microbiome according to the three most probable climatic scenarios established by the IPCC (AR5), using a dynamic model based on Island Biogeography. The dataset is unique to explore the temporal dynamics of the long-range dispersal of airborne bacteria, fungi, and protists (i.e., aeroplankton).

## Materials and methods

### Data

Briefly, aeroplankton samples were collected fortnightly, from May 15, 2007, to October 15, 2013, at the protected area of the Aigüestortes i Estany de Sant Maurici National Park (42° 33′ N, 0° 53′ E; the Pyrenees, northeastern Spain)^14^. We collected washed out aerosols in wet deposition (both rain and snow) at high elevation (ca. 1700 m.a.s.l.), height that for our location is proposed to be above the boundary layer (ABL)^17,18^. We further checked this assumption using NOAA HYSPLIT models and their metadata for the 688 wet deposition events that were collected in the present study, and confirmed that the mean for the boundary layer height was below 1500 m.a.s.l. for all months along our samplings. Collecting wet depositions ABL can reduce the influence of the near-surface aerosols in comparison to air dynamic sampling, and has been proposed as useful, cost-effective proxies, for monitoring the long-term intercontinental exchange of high-atmosphere airborne microorganisms^19^. Although microorganisms recovered by wet collectors may not represent the full composition of the tropospheric air community, due to differential separation in processes such as aerosolization, transport, or scavenging^20^, wet depositions can account for a major amount of the total deposition^21^.

DNA was extracted using the Mobio PowerSoil DNA Isolation Kit (Mobio Laboratories). We carried out PCR and high-speed multiplexed SSU rRNA gene Illumina MiSeq sequencing for 16S and 18S rRNA genes, and taxonomically assigned OTUs (97% identity). Potential pathogenic microbes were identified and catalogued previously (Triadó-Margarit et al.^22^; see Supplementary Material for more information). A total of 112 and 111 samples of bacterial and eukaryal communities, respectively, were considered in this study. Previous work has already used partially or completely this dataset^14,19,22–24^.

Climatic variables associated to the samples, such as daily mean, maximum, and minimum temperatures (Tmed, Tmax, and Tmin respectively), together with humidity (Hum.) and irradiance (Irr.), were recovered from a nearby weather station (Boi, 2535 m.a.s.l.). Local meteorological conditions reflected the meteorological history of the tropospheric transport path for all precipitation events, which was obtained from the NOAA HYSPLIT models. We also measured wet deposition volume (rain hereafter) and chemical variables, indicative of the origin of the air-masses^14^, such as acid-neutralizing capacity (ANC), conductivity (Cond.), dissolved inorganic carbon (DIC), dissolved organic carbon (DOC), total nitrogen (TN), or total phosphorus (TP), and sampling effort (Samp. Eff.). Supplementary Information includes a full list of chemical variables and further details on data acquisition, manipulation, and analysis.

### Temporal dynamics

We used a dynamic model based on Island Biogeography to follow the temporal evolution of aeroplanktonic richness^25^. Two basic mechanisms control model dynamics, colonization (the addition of new detected species to the airborne community) and extinction (the loss of species detection in the airborne community). The thoroughness of this longitudinal dataset allowed for the application of the model to the aeroplanktonic community^24^. Thus, we estimated these mechanisms and the influence of environmental variables on them^26^. We assumed a linear response of colonization and extinction to the studied environmental variables. Model parameters for each taxonomic group were fitted using a greedy algorithm^26^, that selected sequentially the environmental variable (either climatic or chemical) that explained better the observed temporal dynamics. The same was done for the most diverse groups of potential pathogens, i.e. human and plant pathogenic fungi. For more details on the models, see Supplementary Information.

### Prediction of microbial responses to climate change

We followed several steps to predict the response of the airborne microbiome to climate change. The first step was to predict future temperatures, which are readily available and per se important in the dynamics of the different groups. We downloaded regional climate models that predicted temperatures (minimum, average, and maximum) from 2001 to 2100 for three different Representative Concentration Pathways (RCP), RCP2.6, RCP4.5, and RCP8.5, from EUROCORDEX. The different RCP scenarios are named after their relative radiative forcing against the baseline of 1750. RCP2.6 represents a forcing of 2.6 W/m^2^, a scenario with intense mitigation and future negative emissions of CO_2_. RCP4.5 corresponds to an intermediate scenario with stabilization at 4.5 W/m^2^. RCP8.5 is the high emissions scenario, with a radiative forcing of 8.5 W/m^2^ and CO_2_ levels increasing even after 2100. From the ensemble of regional climate models, we obtained predicted monthly temperatures. We calibrated these temperatures with the ones obtained at the nearest meteorological station. We did not use other climatic variables in this step as they are either not available or are no expected to change in the future, as it is the case of precipitation^27^.

The second step was to obtain sets of future environmental variables. We identified which physicochemical variables of the atmospheric depositions correlated with the actual temperatures of that day. All variables except two (Rain and TP) correlated with temperature. Next, we found the generalized linear model of the different temperatures and their interactions that best explained (lower AIC) each environmental covariate (Table S4). We used these models, with the future temperatures, to obtain predicted values of the physicochemical variables, acknowledging the relationship between temperature and air-mass circulation patterns. We added a random residual extracted from the distribution of residuals of each model. Thus, we obtained values resembling the observed series for each variable. In the case of Rain and TP, future values for these variables were chosen at random from the distribution of observed values. Specifically for TP, we detected seasonality, so we obtained the values at random from its seasonal distribution. The results of this procedure were then assumed as the future values for the environmental variables. We avoided unwanted biases and obtained more reliable estimates of future environmental conditions by repeating 100 times this procedure.

Finally, we predicted species richness for each microbial group. We simulated their dynamics fortnightly for the period 2021–2100, with the previously estimated expressions for colonization and extinction and each set of future environmental conditions, using functions rates_calculator, cetotrans, and data_generation of R Package ’island’^26^. We repeated this procedure 100 times for each replicate of the future environmental conditions. See Supplementary Information for further details.

## Results

**Figure 1:**
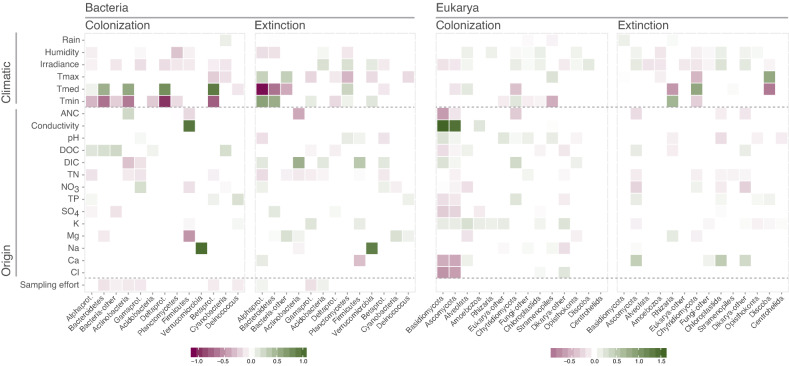
Effect of environmental variables associated with climate or origin (inferred from chemical variables) on colonization and extinction dynamics. The effect is weighted by the logarithm of richness and relative to either colonization or extinction independent terms. Positive values indicate an increase in colonization (or extinction) when a given environmental variable increases.

The temporal dynamics of bacteria were strongly related to climatic factors such as temperature, irradiance, and humidity (Fig. 1). Overall, bacterial dynamics reacted coherently to changes in minimum and mean temperatures. Moreover, the colonization of the richest bacterial groups (i.e., Alphaproteobacteria and Bacteroidetes) increased with DOC enriched atmospheric depositions (rain and snow). In contrast, the origin of aerosols according to the chemical composition of depositions shaped the dynamics of the most abundant and richest eukarya (Fig. 1); ANC, conductivity, Ca^2+^, and Cl^−^ strongly influenced the colonization of fungi Basidiomycota and Ascomycota. Temperature fluctuations also determined the dynamics of other abundant Eukarya such as fungi Chytridiomycota. Rain had scarce effects for both Bacteria and Eukarya. Model parameters and richness for each group are shown in Tables S1 and S2.

**Figure 2:**
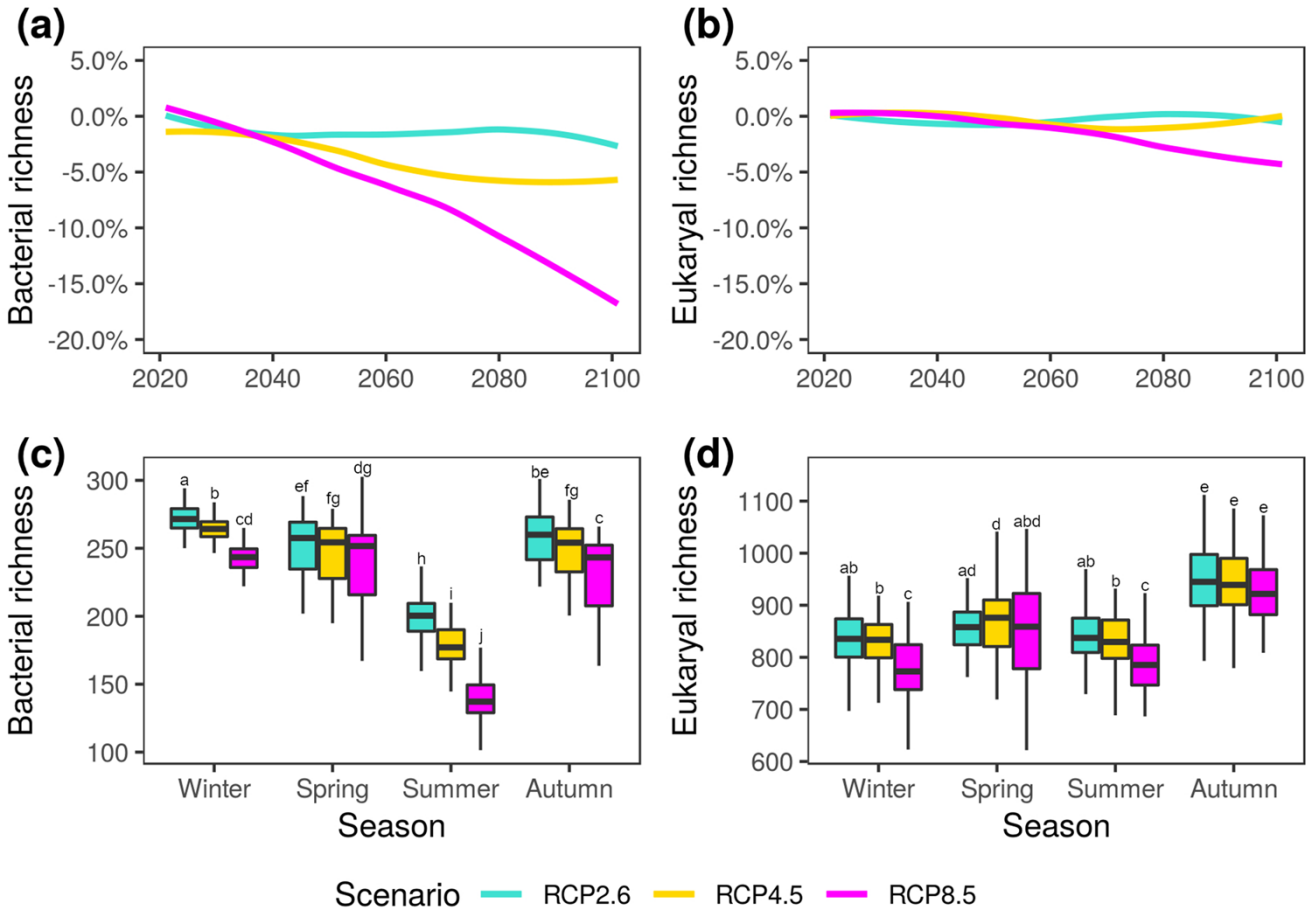
Microbial richness predicted in three different climatic scenarios (RCP2.6, 4.5, and 8.5), corresponding to emissions decline, stabilization, or increase. (**a**) Bacterial or (**b**) eukaryal relative richness change for the period 2020–2100. (**c**) Bacterial or (**d**) eukaryal richness for seasons within the period 2081–2100. The boxes are delimited by quartiles Q1 and Q3, whiskers correspond to 1.5 interquartile ranges from the corresponding quartile, and letters indicate between-group differences based on post-hoc analysis.

Temperature was the key factor for the prediction of airborne microbiome dynamics, either directly (Fig. 1) or through its relation with the remaining environmental variables considered in this study. The ensemble of downscaled regional climate models indicates steady temperature increases up to 5 °C in the worst-case scenario, RCP8.5, and slight increases in the other two scenarios (Fig. S1). We expect changes in the airborne microbiome, with a larger impact as the temperature anomaly gets higher (Fig. 2a,b). In the worst-case scenario, we found that bacterial richness may decrease more than 15% by the end of the century, while eukaryal richness would be stable until 2060 and then decrease slightly. The changes in bacterial groups may be uniformly distributed (Fig. S2). In contrast, the changes in eukarya may affect mainly the richest groups, producing stable richness due to replacement by other groups (Fig. S3). For example, the richness of airborne Alphaproteobacteria and Actinobacteria would decrease over 15%, with a lesser decrease in Bacteroidetes, and higher stability in Betaproteobacteria. Conversely, for eukaryal groups we predict > 30% increase for Alveolata, whereas stability for Chytridiomycota, and decreases for Basidiomycota and Ascomycota are expected. The intermediate scenario, RCP4.5, shows only small changes for Bacteria, and almost no change for Eukarya, for both the whole communities and the specific groups.

We noticed that richness changes were not equally distributed seasonally and got amplified towards the end of the predicted scenarios (Figs. S4, S5). Consequently, the period 2081–2100 was rigorously explored (Fig. 2c,d). For Bacteria, the richness loss in winter and summer is more pronounced, even in the RCP4.5 scenario, also for the abundant groups Actinobacteria, Alphaproteobacteria, and Bacteroidetes, while for Betaproteobacteria we predict slight spring increases (Fig. S6). The losses were almost equally distributed again. In the case of Eukarya, winter and summer displayed more richness loss, while Spring and Autumn remained at the same levels in the worst-case scenario. Ascomycota and Basidiomycota shaped this trend, although Alveolata tends to increase and Chytridiomycota to be somewhat stable (Fig. S7). The changes were negligible in the intermediate scenario. In summary, we expect a more apparent seasonality for both Bacteria and Eukarya if the worst-case scenario is confirmed.

**Figure 3:**
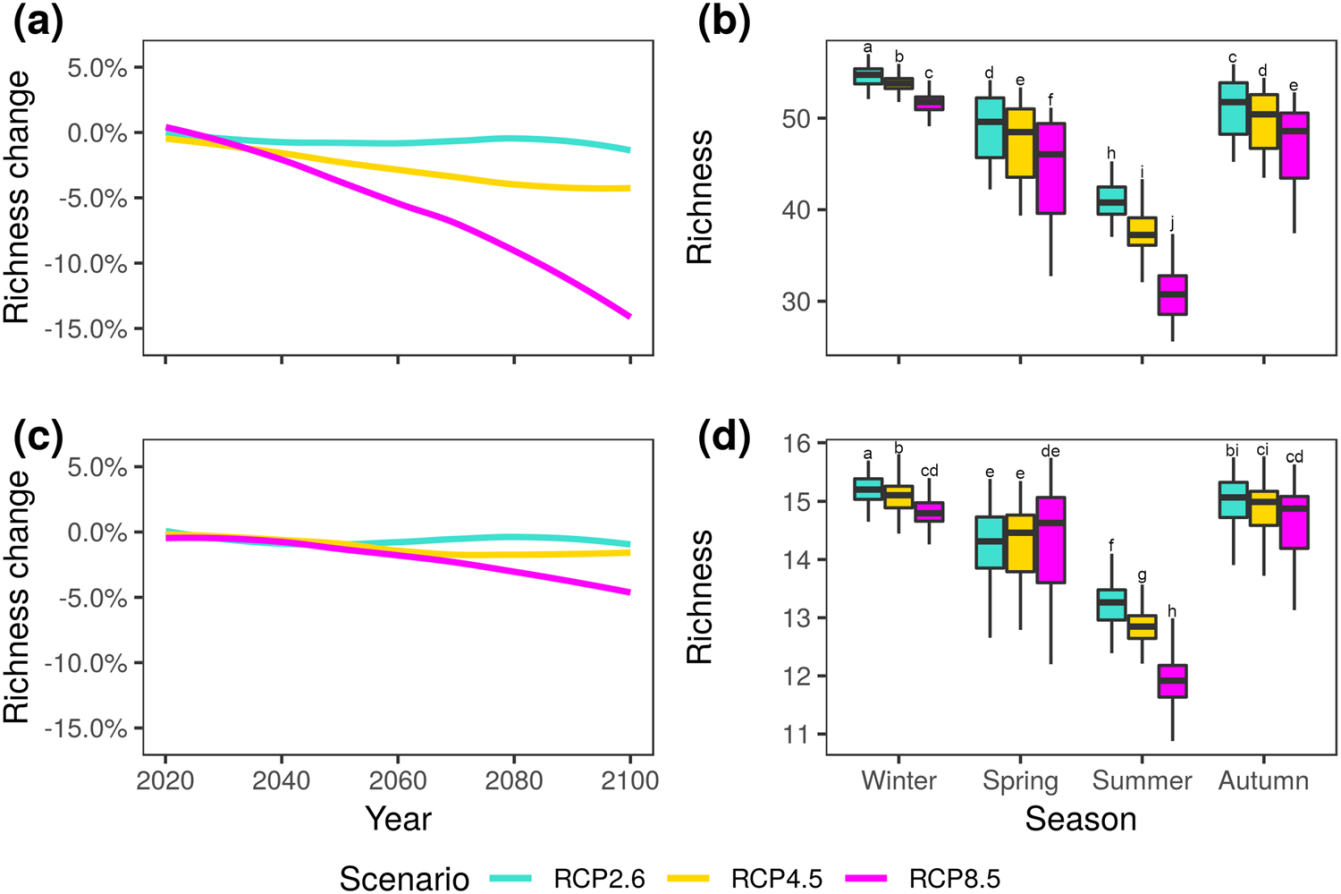
Richness trends and seasonal predictions for plant and human putative eukaryal pathogens. (**a**) Richness change for plant pathogens. (**b**) Seasonal prediction for plant pathogens in 2081–2100. (**c**) Richness change for human pathogens. (**d**) Seasonal prediction for human pathogens in 2081–2100. Letters indicate between-group differences based on post-hoc analysis.

We repeated the same procedure to predict the behavior of potential pathogens. We focused on the specific case of plant and human eukaryal (fungi) pathogens, as those are among the richest pathogen groups (Fig. 3, Table S3). We extracted from the catalogue of potentially pathogenic representatives 120 OTUs (see Supplementary Data for details on the identity of these potential pathogens) that were related to 32 different inventoried plant pathogens, such as *Phoma destructiva* (21 OTUs), *Ophiosphaerella herpotricha* (20), *Peyronellaea pinodella* (15) or *Taphrina padi* (12). These putative eukaryal plant pathogens (mostly fungi), contrary to the remaining eukarya, were more affected by climate than by the aerosol origin. The main drivers for the dynamics of this group were Tmin, Tmax, SO_4_, and Cl^−^. The model predicted that the richness of this group might decrease up to 15% in the worst-case scenario and about 5% in the intermediate scenario, with no decrease in the RCP2.6 scenario. These losses were higher in summer and winter. In the case of putative human pathogens, we considered 32 potentially pathogenic OTUs (see Supplementary Data for details) related to 10 inventoried human pathogens, such as *Coccidioides posadasii* (11 OTUs), *Cryptococcus neoformans* (7) or *Cladosporium cladosporioides* (4). The drivers of their dynamics were TP, Tmin, K, TN, and Mg. The model predicted a slight decrease in the richness of this group in the worst-case scenario, especially in summer, while in more benign scenarios, minor differences were expected.

## Discussion

In this work, we used a simple approach based on ecological theory to predict species richness dynamics in the future airborne microbial communities under three different climate change scenarios (RCP2.6, RCP4.5, RCP8.5). We found richness and composition changes in the airborne microbiome. As far as we know, this is the first prediction exercise on the fate of aeroplankton under future climate change scenarios. In the following paragraphs, we discuss the main findings and examine potential consequences for biological communities and ecosystem processes.

Interestingly, climatic variables affected Bacteria more than chemical variables, while the opposite was found for Eukarya. This result reflects the susceptibility of bacterial cells to atmospheric conditions^16,28^, while eukaryotic cells may mainly correspond to dispersal forms under no additional selective pressures^13^. Based on these findings, we simulated the outcome of future climatic scenarios. Bacterial richness decreased more in the severe scenario. Conversely, eukaryal richness remained stable, but the richest groups, the fungi Ascomycota and Basidiomycota, decreased. These two fungal groups are known to discharge their ascospores and basidiospores under humid conditions^29^. Eukaryal plant pathogens, mostly fungi, also decreased. SAR-Alveolata, which dominate arid and semi-arid regions, increased. Taken together, Eukarya are probably more affected by changes in the origin of the aeroplankton due to predicted changes in the air mass circulation patterns.

Seasonality is a feature of the airborne microbiome^14,16,30^. Our baseline (RCP2.6) predictions of richness are compatible with the seasonal richness reported by^14^, only with minor changes (higher autumn richness for Eukarya). We also confirmed the dominance of groups as Bacteroidetes in winter and spring^30^, although we could not confirm other observations (e.g. the dominance of Alphaproteobacteria in summer). In the worst-case scenario (RCP8.5), many of the seasonal trends found would change, e.g., spring would be the richest season for Bacteria or winter and summer would present the lowest eukaryal richness. These changes would tend to affect equally all bacterial groups, which reflects again their increased susceptibility to the harsh environmental conditions prevailing in the high atmosphere, as a strong environmental filter^28^. However, the changes would hit harder the richest eukaryal groups throughout all seasons, probably reflecting the change in origin and air circulation patterns that increasing temperatures suggest. The consequences of these seasonal changes threaten diversity as complex underlying associations might be altered^31^.

Many predictions of climate change for ecological communities imply loss or interchange of species. The effects on other communities and ecosystem processes are complex to predict, especially in the field of bioaerosols, where knowledge gaps are still huge^9,16^. Some potential consequences may affect cloud formation, remote invasions in sentinel environments, or pathogen dispersal. In the first case, Bacteria have been shown to help the initial development of clouds, although it is unclear whether species identities and physiological status influence this process^32^. Here, we expect lower bacterial richness in aerosol depositions. Together with an expected increase in aerosol loads, it may reduce the diversity and, consequently, alter the effects of the airborne microbiota on cloud formation. Remote alpine lakes are especially suited for ecological research and act as sentinel environments^33^. Airborne microbes have the potential of colonizing these pristine lakes, even migrating intercontinentally^23^. However, the importance of these invasions may change as increased temperatures are expected to decrease microbial richness (this study) and affect composition^30^ in airborne communities. The influence of the aeroplankton on lacustrine ecosystems, also affected by increasing dust loads in the atmosphere, might spread in the entire freshwater network due to priority effects^34^.

Interestingly, plant–microbe interactions may also be affected. Although the influence of airborne communities on leaf communities has been shown experimentally^35^, its importance in natural communities is unclear^7^. Further work is needed to evaluate the effects of changes in the airborne microbiome over the phyllosphere. Additionally, the role of airborne microbes as plant pathogens^5^ might be shaped by future climate. Our prediction for fungal pathogens of plants indicates a sharp decline in richness for the RCP8.5 scenario, particularly in winter and summer, which might suggest that the atmosphere and its selective pressures may represent for them an environmental filter. However, previous studies suggest that the importance of aeroplankton, including airborne bacterial food pathogens, may increase due to higher dust activity and faster winds associated with heavy storms or cyclones^36^.

Overall, the fate of airborne microbiome, and particularly pathogens, under global change^9^ is a key unsolved question yet, and further mechanistic approaches are needed to improve predictions which should inspire robust and proactive measures that minimize risks^36^. Here we proposed dynamic mechanistic models based on ecological theory and previous knowledge to start tackling this endeavour, fitted against one of the most comprehensive aeroplankton studies to date. However, our methods may have several shortcomings. First, we may not cover all processes influencing aeroplankton communities. Nevertheless, our method includes, at least in an effective manner, three of the four fundamental classes of processes acting in ecological communities^37^, dispersal, selection, and drift, and the fourth, speciation, has typically longer timescales than the rest. Second, changes in these processes might skew our predictions. Yet the general responses found seem consistent with previous knowledge and unlikely to change excessively. And third, future conditions may change in a different manner as we have predicted, for example, due to global change, adding uncertainty to our predictions. However, we believe that our method is robust as we consider replicates of different climatic scenarios.

Despite its exploratory nature, our study offers insight into the temporal dynamics of the airborne microbiome. The contrasting importance of the atmospheric environmental filtering and the origin of the aerosols emerged as determinant of the fate of the bacterial and eukaryal communities. Our simulations showed apparent modifications of the airborne microbiome for three future scenarios that might serve as new hypotheses/warnings to be tested in future research. The development of mechanistic models based on ecological theory, together with high-quality, comprehensive microbial datasets and transdisciplinary work, may help us cope with some of the ecological challenges that lie ahead at the dawn of the twenty-first century.

## Supplementary Information

Supplementary Information 1.

Supplementary Information 2.
