# Supplementary material for: General decline in the diversity of the airborne microbiota under future climatic scenarios
Source: Sci Rep. 2021 Oct 12;11:20223. doi: 10.1038/s41598-021-99223-x (PMC8511268; doi:10.1038/s41598-021-99223-x)
Supplement: Supplementary file 2 — Supplementary Information 2. [file 41598_2021_99223_MOESM2_ESM.docx]

# Supplementary information for “General decline in the diversity of the airborne microbiota under future climatic scenarios”

## *Scientific Reports*

Vicente J. Ontiveros^1†^, Joan Cáliz^2^, Xavier Triadó-Margarit^2^, David Alonso^1^, and Emilio O. Casamayor^2†^

1 Theoretical and Computational Ecology, Center for Advanced Studies of Blanes (CEAB-CSIC), Spanish Council for Scientific Research, Accés Cala St. Francesc 14, E-17300 Blanes, Spain

2 Integrative Freshwater Ecology Group, Centre for Advanced Studies of Blanes (CEAB-CSIC), Spanish Council for Scientific Research, Accés Cala St. Francesc 14, E-17300 Blanes, Spain

† Corresponding authors: vicente.jimenez.ontiveros@gmail.com / casamayor@ceab.csic.es

This document contains additional information on methods, data analysis, the study of temporal dynamics, and details on the prediction of the response of the airborne microbiome under future climatic scenarios. It also contains supplementary tables and figures.

# Additional methods: data, temporal dynamics, and predictions.

## Data

Washed out aerosols in wet deposition have recently been proven to be a proper method for monitoring the long-term intercontinental exchange of high-atmosphere airborne microorganisms (1). Atmospheric wet precipitation was collected using an automatic wet and dry passive sampler MTX ARS 1010 (MTX, Bologna, Italy). The wet collector remained covered until the hygroscopic sensor was activated. Particles in the wet container were filtered, dried in a laboratory oven and kept in the dark [6]. The volume and chemical composition of water samples was determined. Major cations (Na, K, Ca, Mg) and anions (Cl, SO4, NO3), pH, acid-neutralizing capacity (ANC), dissolved inorganic (DIC) and organic (DOC) carbon were analyzed as described in (2). Additionally, total nitrogen (TN) and phosphorus (TP) was determined. Airflow trajectories are coupled with the chemical characterization of rain/snow samples and indicate that air mass origin drives the seasonality of aeroplankton and affects long-range dispersal of airborne microbes (2).

The entire genetic dataset is available in the National Center for Biotechnology Information Sequence Read Archive under accession no. PRJEB14358. The methods of the Research Technology Support Facility at Michigan State University (<https://rtsf.natsci.msu.edu/>) were followed to perform PCR and high-speed multiplexed SSU rRNA gene Illumina Miseq sequencing of 16S and 18S rRNA genes, regions and primers listed in SI Appendix in (2). For more details on sequence processing, please refer to (2). In the present study, we kept the original OTU table. However, we took into account differences in sampling effort incorporating sequencing depth to the models, as another variable. Sampling effort did not influence the models for Eukarya, while for Bacteria had minor effects for some groups.

The identification of potentially pathogenic microbes was carried out previously (3) in the same way as published for air samples indoors (4) and biofilm and water samples (5, 6). Briefly, BLAST analyses (7) were used to examine representative OTU sequences against an in-house database of obligate and opportunistic pathogens. Only those OTUs having sequence identity values above 98% and the highest BLAST alignment coverage values (threshold set at 90%) to inventoried pathogens were considered for downstream analysis. As stated earlier (4, 5), these results reflect pathogenic potentiality microbes, as more specific analyses are needed to confirm the pathogenic potential, so this catalogue must be taken with caution.

Finally, we checked to what extent the local meteorological conditions of the sampling site reflected the meteorological history of the tropospheric transport path for all the precipitation events. Backward trajectories and climatic variables during the path were estimated using HYSPLIT-model using the GDAS database in *splitr* R package (parameters fixed as in Caliz et al. 2018). The following variables were considered: potential temperature (theta), air temperature, solar irradiance, relative humidity and rainfall. We then estimated the mean value of these variables through the transport path of the precipitation events for each sample. We found that potential air temperature (theta) correlated with local minimum (r = .767, p-value < .001), maximum (r = .728, p-value < .001), and mean temperatures (r = .770, p-value < .001), and local irradiance (r = .340, p-value < .001). Lower correlations were found between air temperature and local temperatures (r ranging .537 - .585, p-values < .001). Local irradiance also correlated with downward solar radiation flux (r = .644, p-value < .001). On the contrary, neither local humidity nor rainfall correlated with their counterparts or temperatures estimated for the air transport path. However, air humidity correlated with potential temperature in the air transport path (r = -.734, p-value > .001).

## Temporal dynamics

Our methodology to estimate air microbiome dynamics is based on the simplest stochastic model of Island Biogeography (8). This dynamic model indicates that the change in richness in a site follows the next equation:

$$\frac{dS_{S}}{dt}=c \left( S_{P}- S_{S} \right)-e S_{S}$$

where $S_{S}$ corresponds to the OTU-richness in the site, $S_{P}$ to the number of species in the regional pool (i.e., all OTUs identified), and c and e correspond to colonization and extinction rates, respectively. (9) solves the above equation for a single species and determines the associated Markov chain between the two states that each OTU can display in each sample, absent or present, and the transition probabilities between them. Thus, we have the expressions:

$$T_{10}= \frac{c}{c+e}\left( 1-\exp\left( -\left( c+e \right)dt \right) \right); T_{01}= \frac{e}{c+e}\left( 1-\exp\left( -\left( c+e \right)dt \right) \right);$$

for the transition probabilities corresponding to colonization ($T_{10}$) and extinction ($T_{01}$).

Assuming species equivalence and independence, (9) proposes a likelihood method to estimate these colonization and extinction rates from a temporal series of presence-absence of community data. For a regular sampling scheme, with equally spaced samples over time, the likelihood can be defined through the associated Markov chain. Thus, the likelihood of a given data set under colonization and extinction dynamics corresponds to:

$$P\left( M | c, e \right)= {(1- T_{10})}^{N_{00}}{T_{10}}^{N_{10}} {T_{01}}^{N_{01}} {(1- T_{01})}^{N_{11}}$$

where $N_{00}$ is the number of events of repeated absence, $N_{10}$ events of colonization, $N_{01}$ events of extinction, and $N_{11}$ events of repeated presence. The method allows to estimate analytically c and e in the case of regular sampling schemes, and heuristic and semi-analytical methods are implemented for the case of irregular sampling schemes in the ’island’ R Package (10).

Additionally, these colonization and extinction rates might not be constant and could vary alongside several environmental variables. The easiest way to model this dependency is:

$$c_{t}= c_{0}+ \sum_{i=1}^{F} \alpha_{i}Y_{it}; e_{t}= e_{0}+ \sum_{i=1}^{F} \beta_{i}Y_{it};$$

where $Y_{it}$ represents the value of the environmental variable $Y_{i}$ at time t. The coefficients $\alpha_{i}$ and $\beta_{i}$ can be estimated with a greedy algorithm included in R Package island (10), using function greedy_environmental_fit.

We estimated the influence environmental variables separately for those groups of abundant taxonomic ranks in (2) (>1% relative abundance; 12 and 13 groups for bacteria and eukarya, respectively), while the rest of ranks (<1% relative abundance) were included in the same group named as "other" bacteria or eukarya. For the subset of OTUs identified as pathogens, we separated them based on their putative host instead of the taxonomic ranks and considered the influence of the environmental variables within each host.

Sequencing depth is considered as a proxy of sampling effort in microbial ecology. To avoid the influence of different sampling efforts among samples, we included it in the models that considered environmental effects, as another environmental covariate.

Overall, the models presented a good fit of the observed dynamics for both Bacteria and Eukarya, and the groups included in them (Figures S8–S10). We used $R^{2}$ as a means for validation. $R^{2}$ is defined as $1- \frac{\epsilon^{2}}{\epsilon_{0}^{2}}$ where $\epsilon^{2}$ represents the mean quadratic error of a simulated model and $\epsilon_{0}^{2}$ the mean quadratic error of a null model of choice. We selected the mean observed richness as our null model. Given so, our $R^{2}$ would represent the relative performance of our dynamic simulations against the static estimator that is the mean value. We also checked the ability of our models to predict unencountered data, estimating them with three-quarters of the temporal series, and simulating the remaining one (Figure S8).

## Prediction of microbial responses to climate change

We downloaded regional climate models from EURO-CORDEX. We selected the simulations with monthly mean, maximum, and minimum temperatures that were bias-corrected and had predictions for three different Representative Concentration Pathways (RCP), RCP2.6, RCP4.5, and RCP8.5, for the European domain at a resolution of 0.11 degrees. Our search gave, as a result, the models MPI-MMPI-ESM-LR-MPI-CSC-REMO2009 (with two replicates that were averaged) and ICHEC-EC-EARTH-SMHI-RCA4. We selected only the prediction at the cell of the grid that corresponded to our study site, obtaining said temperatures. We obtained an ensemble model by averaging the two models, ranging from 2001 to 2100.

We calibrated the temperatures obtained from the regional climate models with the temperatures of the nearest meteorological station, corresponding to Boi (2535 m.a.s.l.). We obtained data of daily mean, maximum, and minimum temperatures (Tmed, Tmax, and Tmin respectively), together with humidity (Hum.) and irradiance (Irr.), for the years studied, 2007 – 2013. We calculated monthly averages, and we estimated the mean and standard deviation of the monthly averages in the studied period (2007 – 2013). Then, we calibrated the ensemble temperatures for the same period, equalizing the mean and standard deviation of these temperatures with the observed ones, using the equation $y_{i}=\bar{x}_{2}+ x_{i}- \bar{x}_{1}\cdot\frac{\sigma_{2}}{\sigma_{1}}$ , where $\bar{x}_{1}$ and $\bar{x}_{2}$ are respectively the means of the ensemble and observed temperatures and $\sigma_{1}$ and $\sigma_{2}$ their standard deviations. Future temperatures, used for prediction, were then transformed accordingly for the period 2021 – 2100.

To obtain simulations of the future conditions of the Pyrenees, we first identified which physicochemical variables of the collected atmospheric depositions correlated with any of the temperature variables, finding that pH, acid-neutralizing capacity (ANC), conductivity (Cond.), Cl, SO4, NO3, Na, K, Ca, Mg, dissolved inorganic carbon (DIC), dissolved organic carbon (DOC), total nitrogen (TN), Hum., Irr., and sampling effort (Samp. Eff.) did it, while Rain and total phosphorus (TP) did not and were subsequently excluded from the following steps. Next, we found the generalized linear model of Tmax, Tmed, Tmin, and their interactions that best explained (lower AIC) each environmental covariate (Table S4). Using these models and the previously predicted future temperatures, we obtained predicted values for the environmental covariates to which we added a random residual, chosen via a random uniform number in the [0, 1] that represented a quantile of the observed distribution of residuals. In the case of the two excluded environmental covariates, the future values of the environmental covariates were chosen as the value given by the quantile associated with a uniform random number, taking into account seasonality for TP, as we did not detect seasonality for Total Rain. The results of this procedure were then the future values for the environmental variables.

## References

1. Triadó-Margarit X, Caliz J, Reche I, Casamayor EO. High similarity in bacterial bioaerosol compositions between the free troposphere and atmospheric depositions collected at high-elevation mountains. Atmospheric environment. 2019;203:79-86.

2. Cáliz J, Triadó-Margarit X, Camarero L, Casamayor EO. A long-term survey unveils strong seasonal patterns in the airborne microbiome coupled to general and regional atmospheric circulations. Proceedings of the National Academy of Sciences. 2018;115(48):12229-34.

3. Triadó-Margarit X, Cáliz J, Casamayor EO. A long-term atmospheric baseline for intercontinental exchange of airborne pathogens. Environment International. 2021:*under review.*

4. Triadó‐Margarit X, Veillette M, Duchaine C, Talbot M, Amato F, Minguillón MC, et al. Bioaerosols in the Barcelona subway system. Indoor air. 2017;27(3):564-75.

5. Subirats J, Triadó‐Margarit X, Mandaric L, Acuña V, Balcázar JL, Sabater S, et al. Wastewater pollution differently affects the antibiotic resistance gene pool and biofilm bacterial communities across streambed compartments. Molecular ecology. 2017;26(20):5567-81.

6. Auguet O, Pijuan M, Borrego CM, Rodriguez-Mozaz S, Triadó-Margarit X, Della Giustina SV, et al. Sewers as potential reservoirs of antibiotic resistance. Science of the Total Environment. 2017;605:1047-54.

7. Altschul SF, Gish W, Miller W, Myers EW, Lipman DJ. Basic local alignment search tool. Journal of molecular biology. 1990;215(3):403-10.

8. Simberloff DS. Experimental zoogeography of islands: a model for insular colonization. Ecology. 1969;50(2):296-314.

9. Alonso D, Pinyol‐Gallemí A, Alcoverro T, Arthur R. Fish community reassembly after a coral mass mortality: higher trophic groups are subject to increased rates of extinction. Ecology letters. 2015;18(5):451-61.

10. Ontiveros VJ, Capitán JA, Arthur R, Casamayor EO, Alonso D. Colonization and extinction rates estimated from temporal dynamics of ecological communities: The island r package. Methods in Ecology and Evolution. 2019;10(7):1108-17.

# Supplementary tables.

**Table S1.** **Models and OTU richness for bacterial groups.** Columns represent the model for each group. Values indicate the coefficient that multiplies the corresponding environmental variable. ANC: acid-neutralizing capacity, Cond.: conductivity, DIC: dissolved inorganic carbon, DOC: dissolved organic carbon, Hum.: humidity, Irr.: irradiance, Samp. Eff.: sampling effort, Tmax: maximum temperature, Tmed: mean temperature, Tmin: minimum temperature, TN: total nitrogen, TP: total phosphorus, c₀: colonization independent term, e₀: extinction independent term. Richness indicates OTU richness for each group, and it is not a coefficient included in the model.

**Table S2. Models and OTU richness for eukaryal groups.** Columns represent the model for each group. Values indicate the coefficient that multiplies the corresponding environmental variable. ANC: acid-neutralizing capacity, Cond.: conductivity, DIC: dissolved inorganic carbon, DOC: dissolved organic carbon, Hum.: humidity, Irr.: irradiance, Samp. Eff.: sampling effort, Tmax: maximum temperature, Tmed: mean temperature, Tmin: minimum temperature, TN: total nitrogen, TP: total phosphorus, c₀: colonization independent term, e₀: extinction independent term. Richness indicates OTU richness for each group, and it is not a coefficient included in the model.

**Table S3.** **Models and OTU richness for two potential pathogen groups.** The eukaryal potential pathogen groups were selected classified according to their potential host. N, richness of potential pathogen groups.

| Group | Colonization | Extinction | N |
| --- | --- | --- | --- |
| Humans | -0,00264 * TP - 0,00428 * Tmin + 0,02523 | -0,00847 * K + 0,00486 * TP - 0,00378 * TN + 0,00315 * Mg + 0,03116 | 32 |
| Plants | -0,00048 * Tmax - 0,00551 * Tmin - 0,00390 * Rain + 0,00377 * Samp. Eff. - 0,00119 * SO4 + 0,03537 | -0,00364 * Cl + 0,03537 | 120 |

**Table S4.** **Relationship between variables and temperature.** Variables related to climate and origin of the aerosol displayed linear relationships with temperature, except rain and total phosphorus. ANC: acid-neutralizing capacity, Cond.: conductivity, DIC: dissolved inorganic carbon, DOC: dissolved organic carbon, Hum.: humidity, Irr.: irradiance, Samp. Eff.: sampling effort, Tmax: maximum temperature, Tmed: mean temperature, Tmin: minimum temperature, TN: total nitrogen.

# Supplementary Figures.


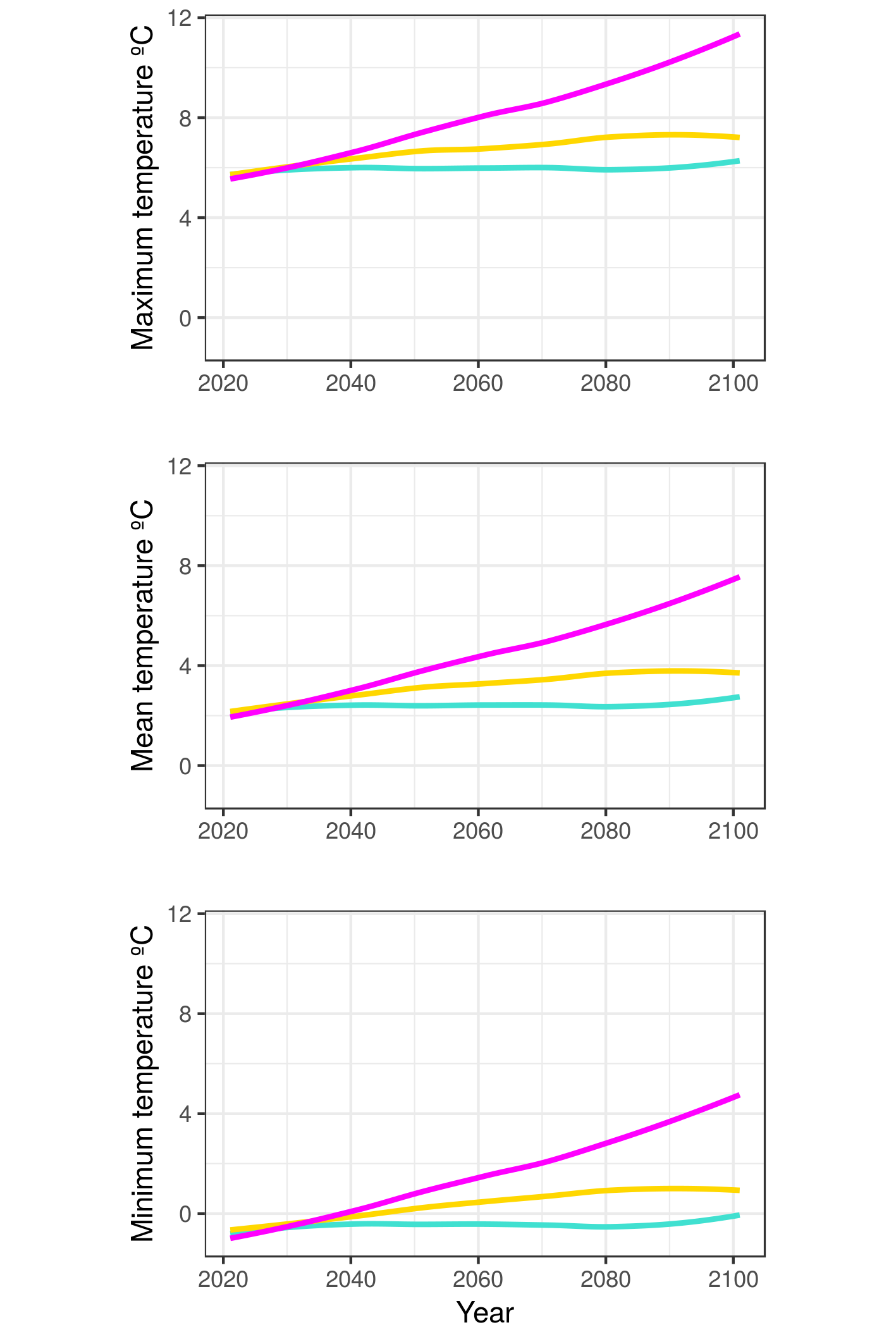


**Figure S1. Predicted increases in temperature.** Predicted increase in temperatures by the ensemble model in the period 2021 – 2100, under three different climatic scenarios (RCP2.6, 4.5, 8.5). Cyan line, RCP2.6, yellow line, RCP4.5, magenta line, RCP8.5.


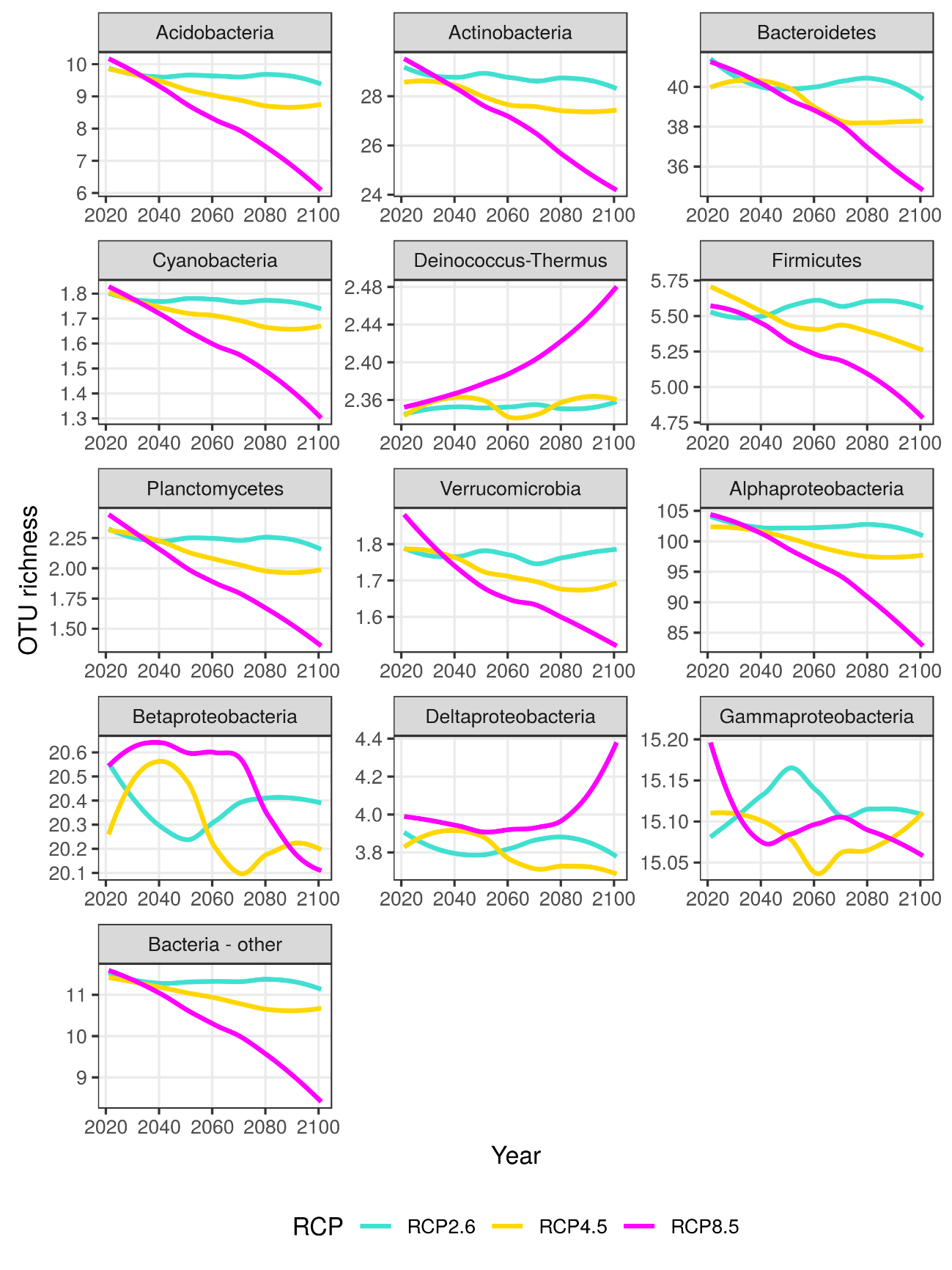


**Figure S2. Richness change for bacterial groups.** Predicted change in OTU richness, under three different climatic scenarios (RCP2.6, 4.5, 8.5).


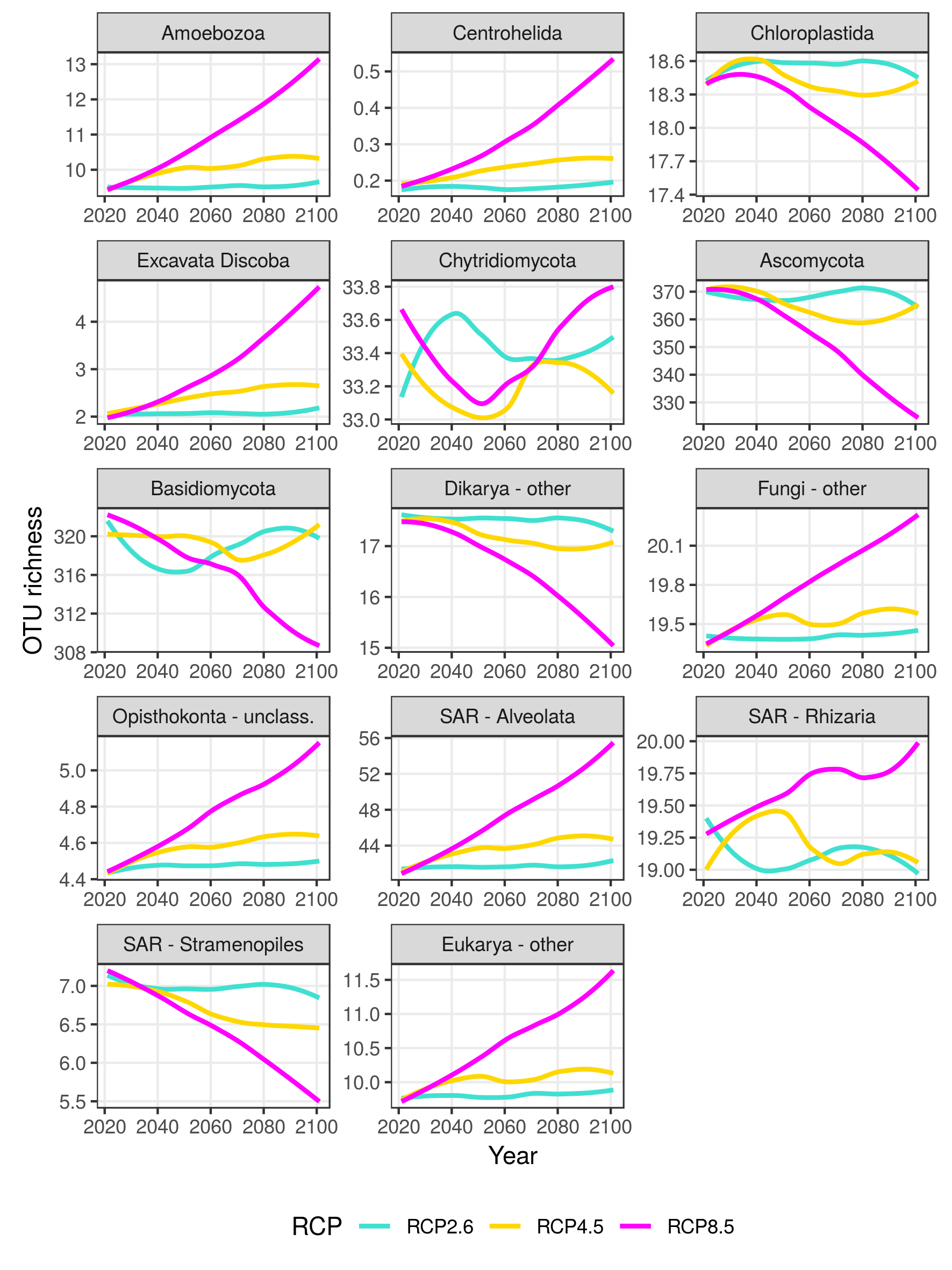


**Figure S3. Richness change for eukaryal groups.** Predicted change in OTU richness, under three different climatic scenarios (RCP2.6, 4.5, 8.5).


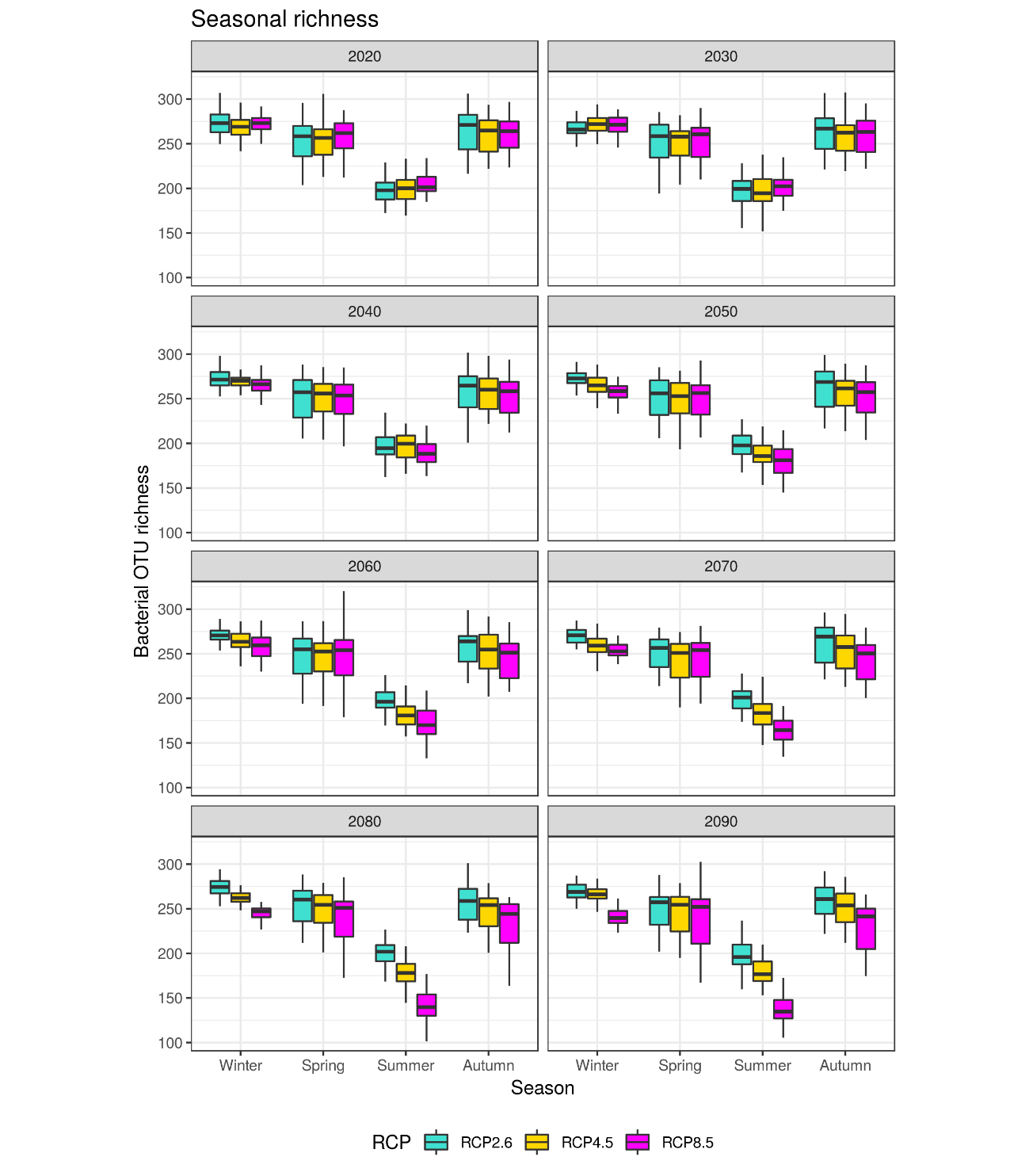


**Figure S4. Predicted seasonal richness for Bacteria.** Predicted seasonal OTU richness for Bacteria, under three different climatic scenarios (RCP2.6, 4.5, 8.5). Changes exacerbate towards the end of the century.


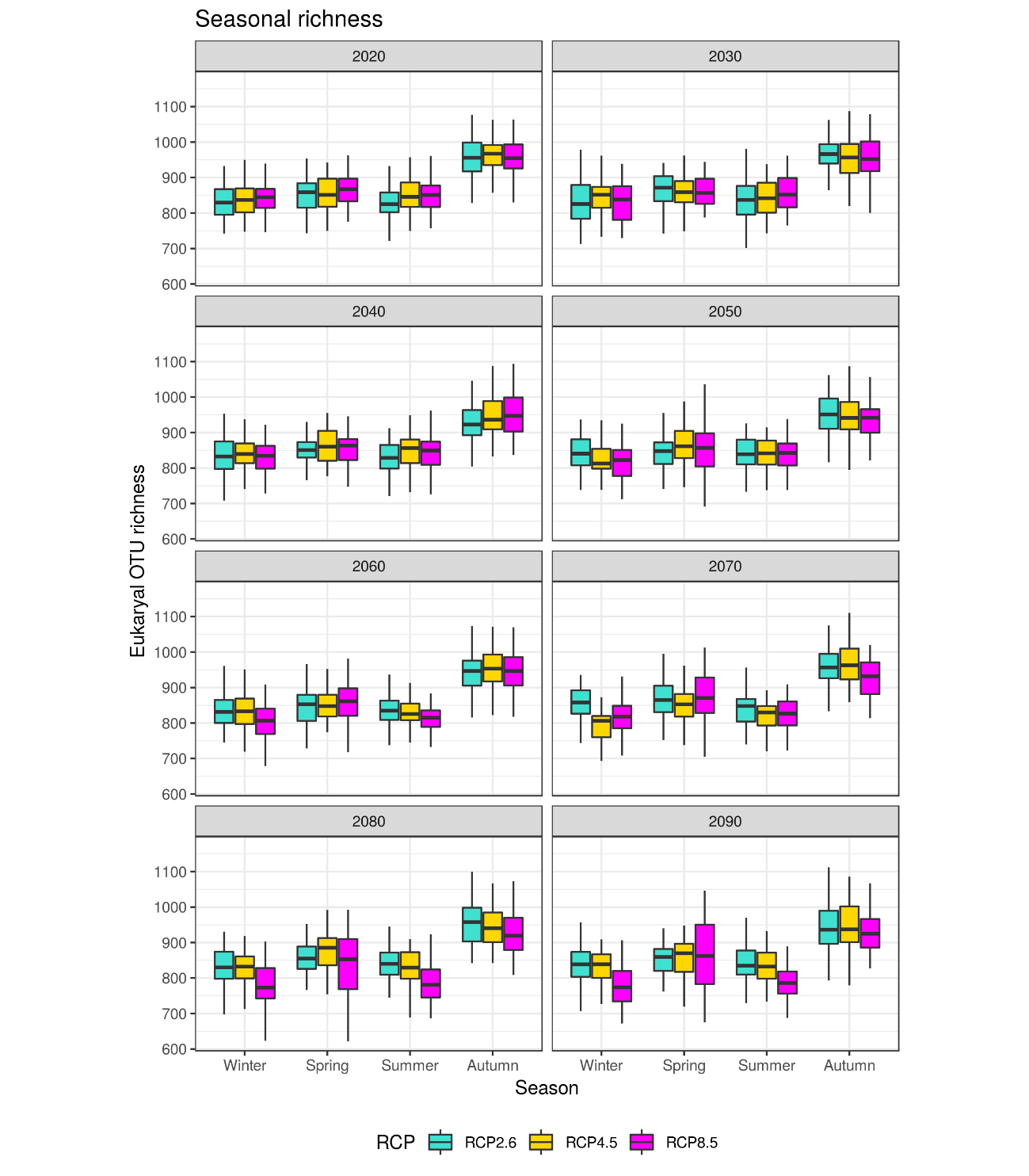


**Figure S5. Predicted seasonal richness for Eukarya.** Predicted seasonal OTU richness for Eukarya, under three different climatic scenarios (RCP2.6, 4.5, 8.5). Changes exacerbate towards the end of the century.


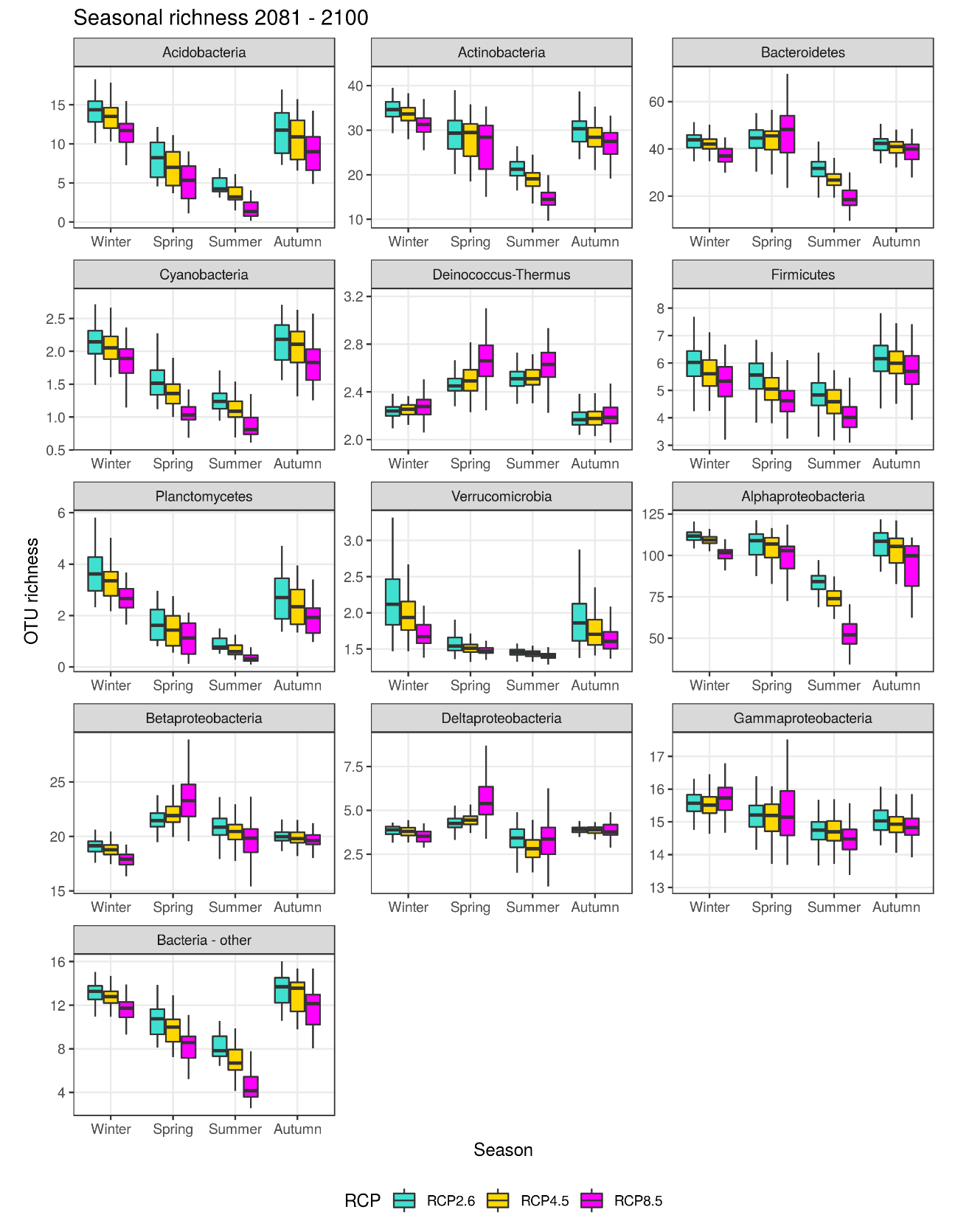


**Figure S6. Predicted seasonal richness for bacterial groups in the period 2081 – 2100.** Predicted seasonal OTU richness for bacterial groups, under three different climatic scenarios (RCP2.6, 4.5, 8.5).


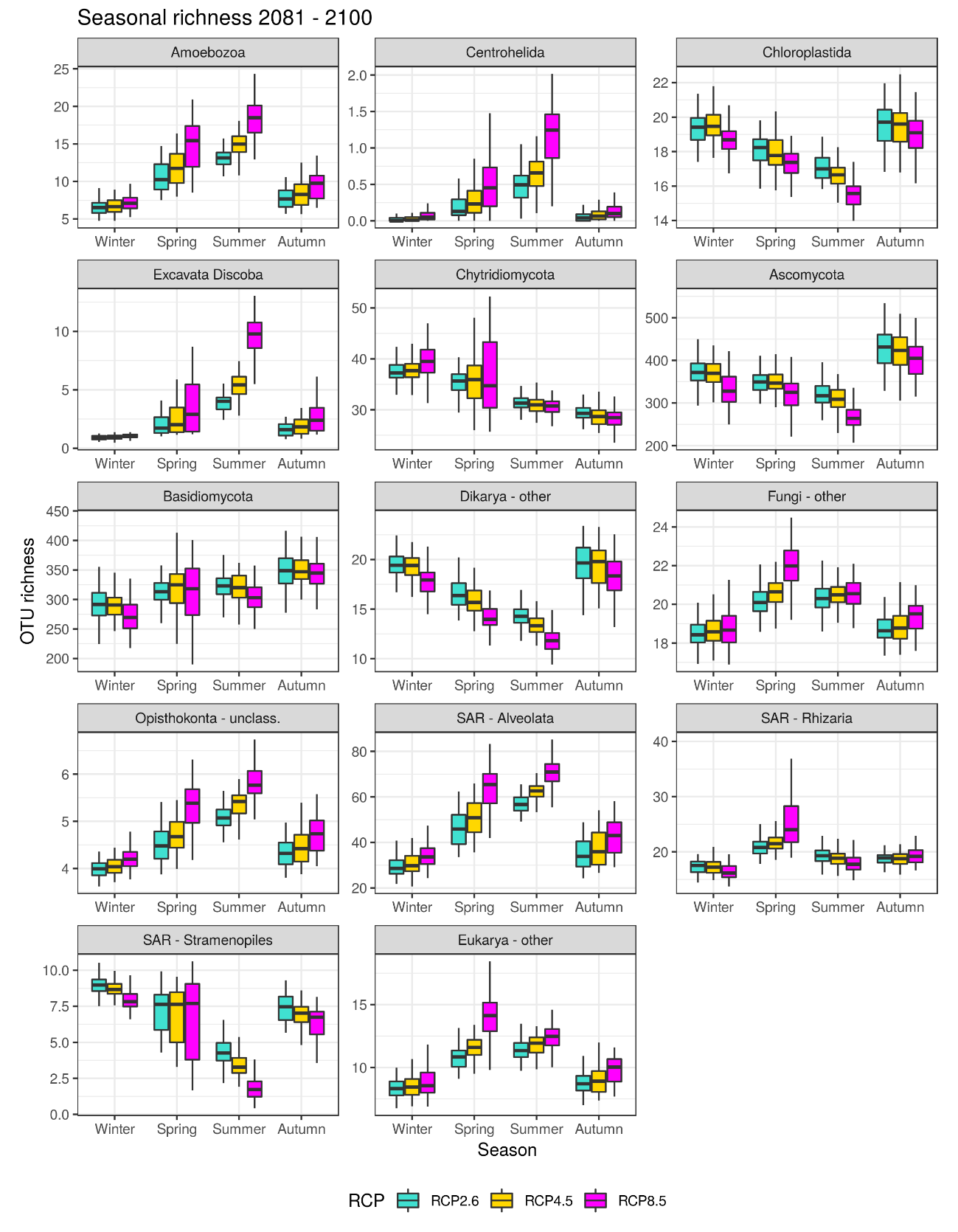


**Figure S7. Predicted seasonal richness for eukaryal groups in the period 2081 – 2100.** Predicted seasonal OTU richness for eukaryal groups, under three different climatic scenarios (RCP2.6, 4.5, 8.5).


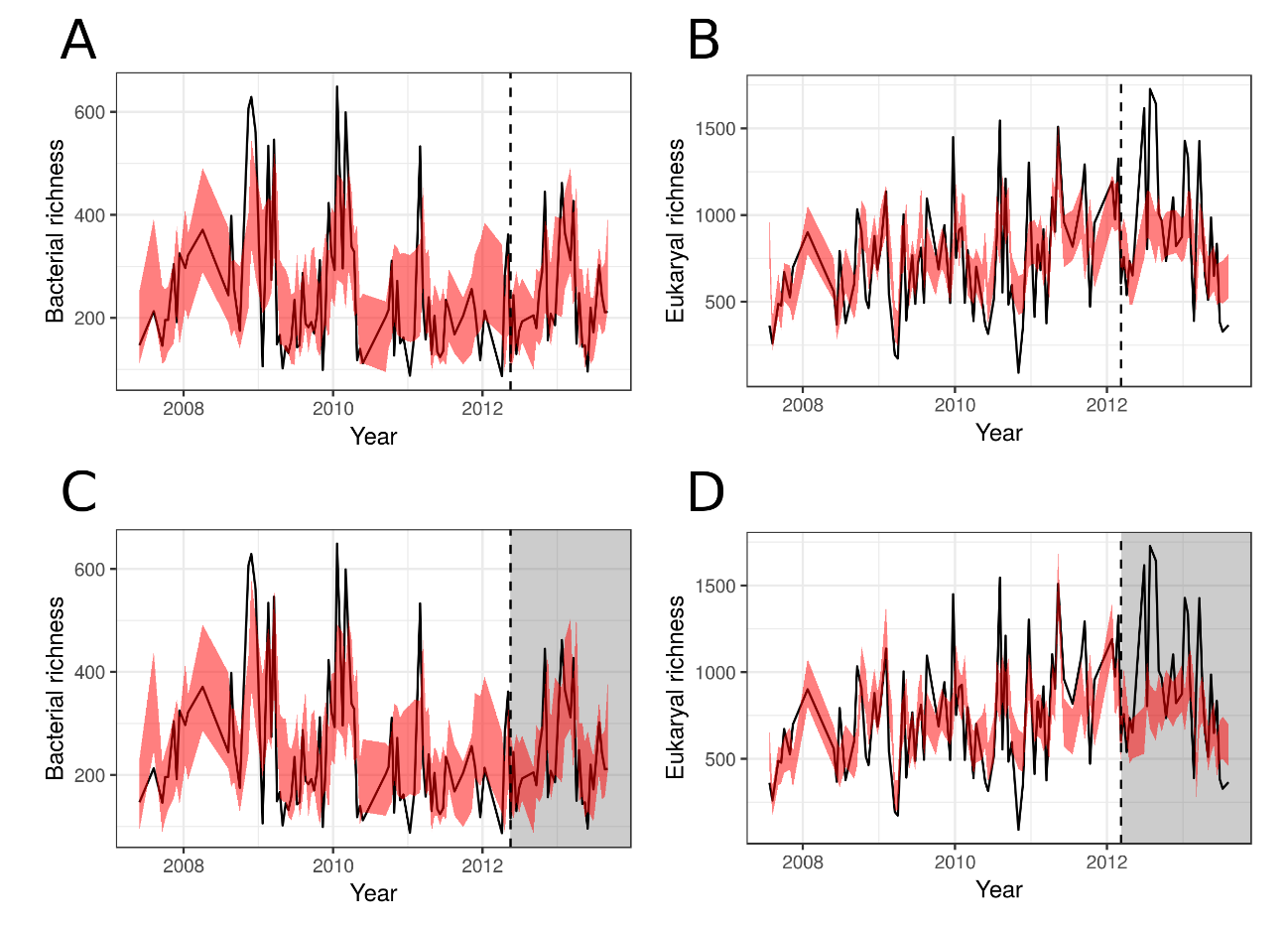


**Figure S8. Validation of the models of temporal dynamics for Bacteria and Eukarya.** Black lines indicate the observed OTU richness. The red ribbon indicates 95% of the simulations. For the whole temporal series, we found an $R^{2}$ of 0.323 and 0.303 for the bacterial (A) and eukaryal (B) model respectively estimated with the whole dataset, while these values descended to 0.324 (C) and 0.233 (D) for the model estimated with three-quarters of the temporal series. The last quarter of the data (grey shade) was used for validating the prediction. There, $R^{2}$ was 0.229 (A) and 0.304 (B) estimated with the whole series, while the validation model performance was as good as the static null model that was the mean, with $R^{2}$ 0.003 (C) and 0.058 (D).


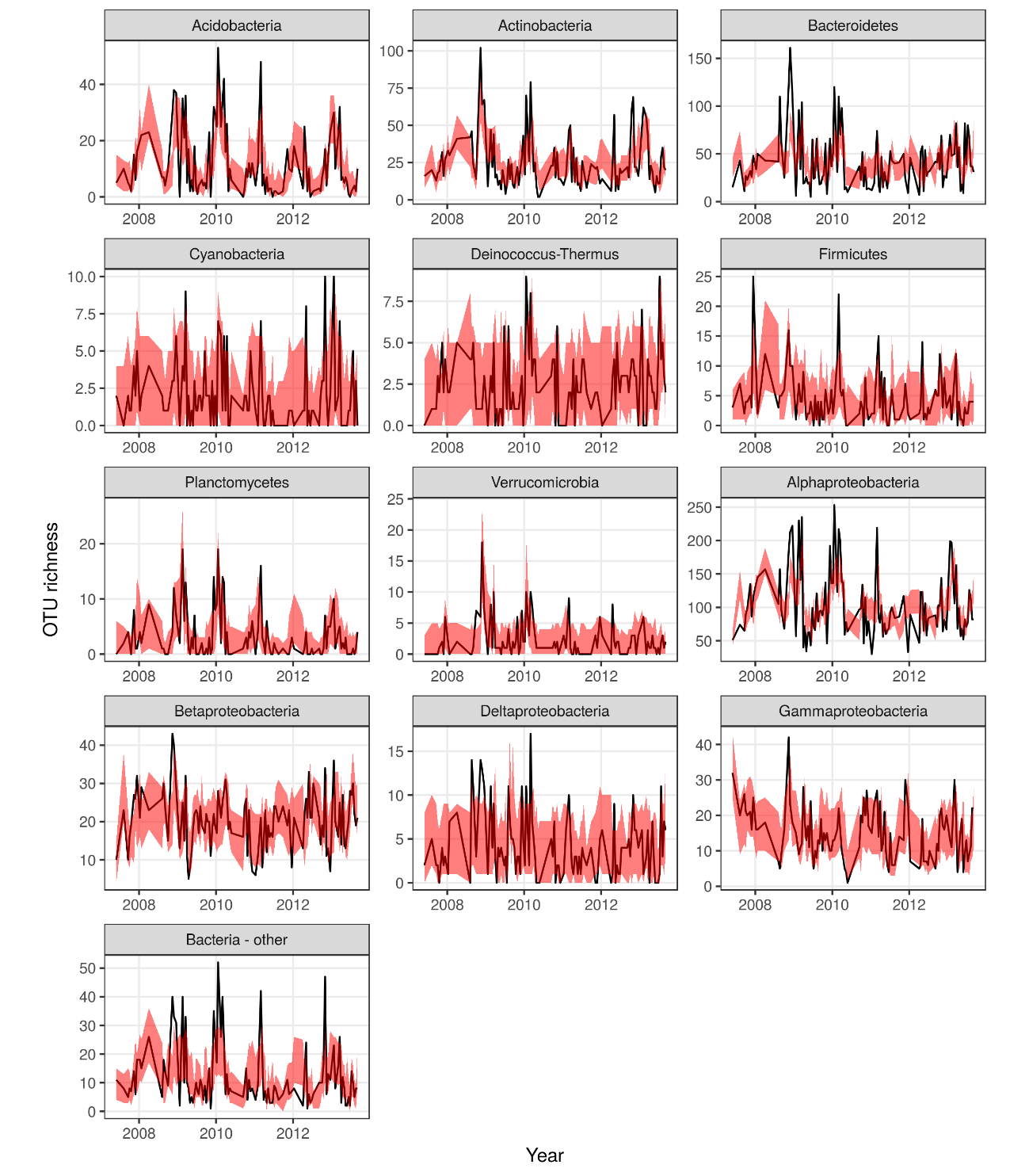


**Figure S9. Observed and simulated OTU richness for bacterial groups.** Black lines indicate the observed OTU richness. The red ribbon indicates 95% of the simulations.


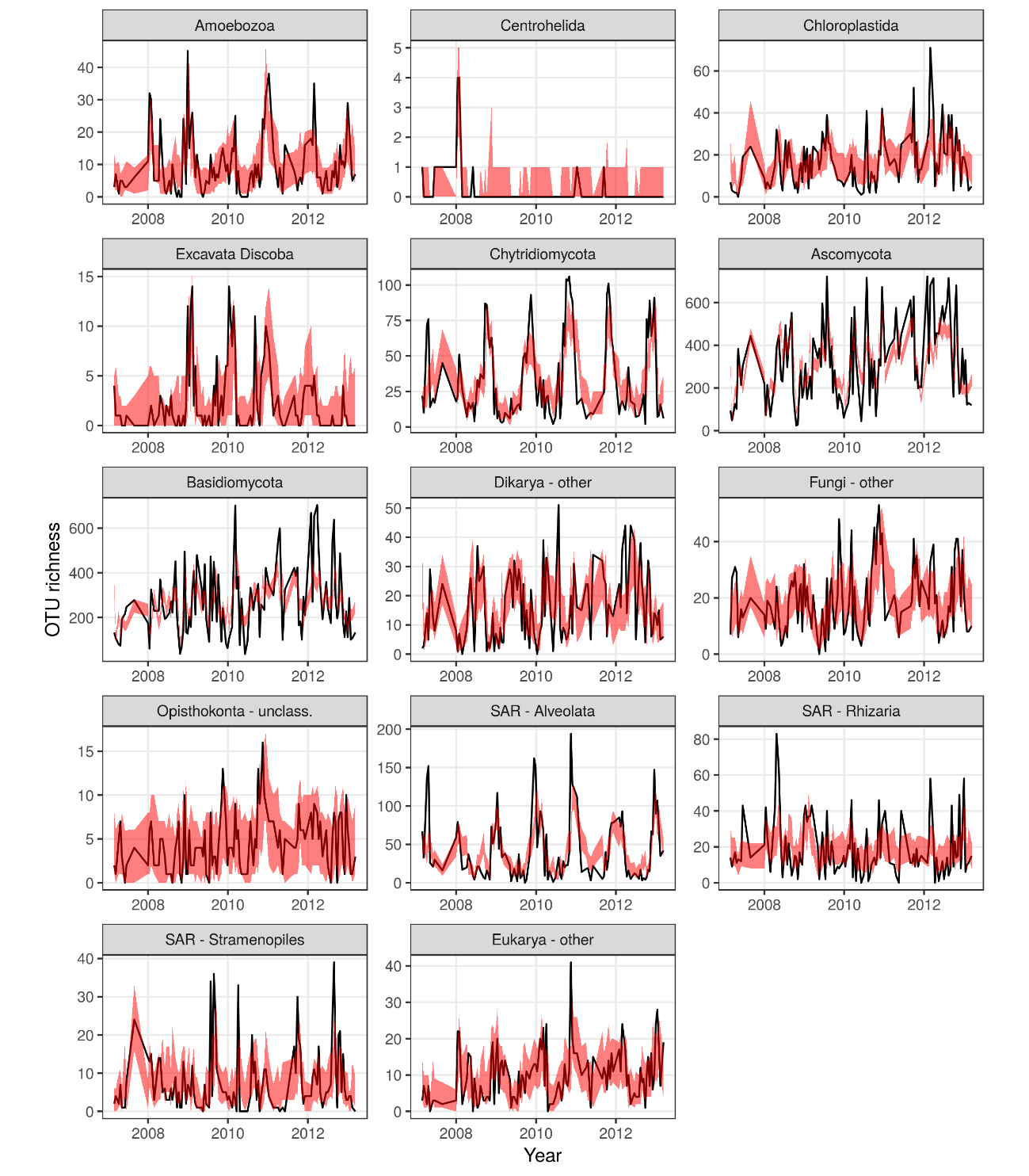


**Figure S10. Observed and simulated OTU richness for eukaryal groups.** Black lines indicate the observed OTU richness. The red ribbon indicates 95% of the simulations.
